# 免疫检查点抑制剂相关肺炎的临床诊治建议

**DOI:** 10.3779/j.issn.1009-3419.2019.10.03

**Published:** 2019-10-20

**Authors:** 汉萍 王, 潇潇 郭, 佳鑫 周, 炼 段, 晓燕 斯, 丽 张, 玥 李, 小伟 刘, 孟昭 王, 举红 施, 力 张

**Affiliations:** 1 100730 北京，中国医学科学院，北京协和医院呼吸内科 Department of Respiratory Medicinev, Peking Union Medical College Hospital, Peking Union Medical College and Chinese Academy of Medical Sciences, Beijing 100730, China; 2 100730 北京，中国医学科学院，北京协和医院心内科 Department of Cardiology, Peking Union Medical College Hospital, Peking Union Medical College and Chinese Academy of Medical Sciences, Beijing 100730, China; 3 100730 北京，中国医学科学院，北京协和医院风湿免疫科 Department of Rheumatism and Immunology, Peking Union Medical College Hospital, Peking Union Medical College and Chinese Academy of Medical Sciences, Beijing 100730, China; 4 100730 北京，中国医学科学院，北京协和医院内分泌科 Department of Endocrinology, Peking Union Medical College Hospital, Peking Union Medical College and Chinese Academy of Medical Sciences, Beijing 100730, China; 5 100730 北京，中国医学科学院，北京协和医院检验科 Department of Clinical Laboratory, Peking Union Medical College Hospital, Peking Union Medical College and Chinese Academy of Medical Sciences, Beijing 100730, China; 6 100730 北京，中国医学科学院，北京协和医院消化内科 Department of Digestive Medicine, Peking Union Medical College Hospital, Peking Union Medical College and Chinese Academy of Medical Sciences, Beijing 100730, China; 7 100730 北京，中国医学科学院，北京协和医院眼科 Department of Ophthalmology, Peking Union Medical College Hospital, Peking Union Medical College and Chinese Academy of Medical Sciences, Beijing 100730, China

**Keywords:** 免疫检查点抑制剂, 免疫相关不良反应, 检查点抑制剂相关肺炎, Immune checkpoint inhibitor, Immunotherapy-related toxicities, Checkpoint inhibitor pneumonitis

## Abstract

免疫检查点抑制剂在肿瘤中的使用给晚期肿瘤患者带来了新的希望。然而，由免疫检查点抑制剂激活的免疫系统，其中主要是T细胞免疫，可能攻击人体正常组织器官导致相应的免疫毒性反应产生，在肺部可引起免疫检查点抑制剂相关肺炎。这是一类不同于已知肺部间质性肺炎的疾病，如处理不当，有潜在的致命风险。我们将对免疫检查点抑制剂相关肺炎的诊断和治疗给予建议。

## Abstract (en)

The increasing use of immunocheckpoint inhibitors in tumors has brought new hope of survival to patients with advanced tumors. However, the immune system activated by immunocheckpoint inhibitors, mainly activated T-cell immunity, may attack normal tissues and organs of the human body and lead to a variety of adverse effects. In the lung, they could induce checkpoint inhibitor associated pneumonitis (CIP). CIP is different from known pulmonary interstitial pneumonitis, and had a potentially fatal risk if it was not being properly treated. We will summarize the characteristics of CIP and give our advice on how to manage immunocheckpoint inhibitor associated pneumonitis.

## 1 概述

由免疫检查点抑制剂（immune checkpoint inhibitor, ICI）治疗引发的检查点抑制剂肺炎（checkpoint inhibitor pneumonitis, CIP）是ICI相关并发症的一种。CIP定义为在患者接受ICI治疗后，胸部影像学出现新的浸润影，临床除外新的肺部感染或肿瘤进展等情况下，出现呼吸困难和/或其他呼吸体征/症状（包括咳嗽和活动后气短等）^[1]^。

临床试验报道的CIP的发生率大多在3%-5%^[2-6]^。Wang等^[4]^最新的*meta*分析了125项研究的18, 715例患者，均使用单药程序性死亡受体-1（programmed cell death receptor-1, PD-1）或PD-L1抑制剂治疗，其中肺炎的发生率为2.79%（95%CI: 2.39%-3.23%），三级以上的肺炎发生率为0.67%（95%CI: 0.50%-0.89%）。另一项针对23项随机对照试验（randomized controlled trial, RCT）研究的12, 876例患者的*meta*分析提示，PD-1抑制剂相关的CIP发生率为5.17%，其中3级-5级CIP的发生率为4.14%；其中帕博利珠单抗3级-5级的CIP发生率似乎更高（5.64%）。相对而言，PD-L1抑制剂的各级及3级-5级CIP的发生率均相对较低（3.25%、2.12%），CTLA-4单药似乎并不增加肺炎的发生，但是和PD-1/PD-L1抑制剂联合时则CIP的发生增加^[5]^。另外，研究显示，CIP的发生率也可能与肿瘤类型有关，其中非小细胞肺癌（non-small cell lung cancer, NSCLC）和肾细胞癌中CIP的发生率高于黑色素瘤^[6]^。然而，临床实验之外，真实世界中ICI治疗肿瘤引起CIP的发生率尚不清楚。回顾性研究^[7]^曾提示，真实世界中ICI引起的CIP发生率显著高于临床实验的报告。而对于中国患者中，CIP的发生率也尚不清楚。

有研究^[2]^分析也提示，PD-1/PD-L1抑制剂治疗相关的总体死亡率为0.45%（82/18, 353），而其中以CIP引起的死亡最为常见（23/82, 28.0%）。由此可见，CIP的总体发生率虽然不高，但是严重的CIP如果处理不当，则可能出现危及生命的严重后果。因此临床医生需要对这一罕见但严重的不良事件给予更多的关注。

临床上，CIP缺乏特异性的临床症状，影像学表现多种多样，缺乏特异性，也缺乏血清学标志物，此外，有时候临床上很难彻底除外感染，特别是病情严重无法进行支气管镜检查者，导致部分CIP的诊断困难。治疗上，大部分CIP对激素治疗敏感，然而仍有15%-30%的CIP对激素反应差，对于这部分难治性CIP，我们缺乏对其病理生理的了解，在治疗上也没有非常好的数据支持，导致这部分患者的预后较差。

## 2 CIP的危险因素及治疗前处理

CIP危险因素目前尚不清楚。人们怀疑性别、高龄、吸烟史、基础肺功能下降、肺手术史、肺部放疗史等因素可能与CIP的发生相关，但是迄今为止尚缺乏有力的依据证明此相关性^[8]^。但基础肺疾病的存在以及基础肺功能的下降，可能导致患者合并CIP时耐受性更差，病情更重，预后更差。多项研究^[3, 7, 9]^均提示。肿瘤类型（尤其肺癌）、使用的ICI种类（PD-1抑制剂发生率较高，PD-L1抑制剂发生率较低）可能与CIP有关。两项回顾性研究^[10, 11]^提示，基础存在的肺间质纤维化可能增加CIP的发生风险。

对于拟接受ICI治疗的患者，建议完善胸部计算机断层扫描（computed tomography, CT）。对于存在基础肺疾病，如慢性阻塞性肺疾病（chronic obstructive pulmonary disease, COPD）、肺间质纤维化者，或平时存在剧烈活动后气短者，应完善肺功能检查。对于女性患者存在基础肺间质疾病者，或影像学提示患者肺间质改变不符合典型的特发性肺纤维化者，建议筛查免疫指标，包括抗核抗体（antinuclearl antibody, ANA)、抗可提取性核抗原（extractable nuclear antigen, ENA）抗体、抗中性粒细胞胞浆抗体（antineutrophil cytoplasmic antibody, ANCA）、类风湿关节炎相关抗体等，除外存在基础免疫性疾病的可能。

对于存在基础COPD者，建议根据肺功能提示，给予充分的吸入支气管舒张剂控制基础肺疾病。对于合并特发性肺间质纤维化者，使用吡啡尼酮、尼达尼布等药物是否可降低CIP的发生目前并无证据。不建议患者预防性使用激素。

## 3 CIP的临床表现

使用ICI后发生CIP的时间范围变化很大，从给药开始到停药后均可出现。临床中应全程对CIP进行监测。但研究^[12]^也提示，早期出现的肺炎可能级别更高。

CIP患者的临床表现具有非特异性，最常见的症状为呼吸困难、活动耐量下降及咳嗽，也可出现发热、胸痛等表现。如出现发热，或后期出现发热，更需要除外感染性肺炎的可能。病程上，CIP可以表现为爆发性、急性、亚急性、慢性以及隐匿性病程等。

CIP患者的体征也缺乏特异性。晚期患者体格检查时可出现肺间质性疾病的相似体征，即中下肺出现爆裂音。部分患者体格检查肺部无明显异常，部分合并感染或心功能不全者可出现湿性罗音。少数患者甚至可出现哮鸣音。

实验室检查方面，血常规可正常或升高（包括白细胞、中性粒细胞等，淋巴细胞计数的改变均无明显特异性）。炎性指标如C反应蛋白、血沉常可升高。其他血清学检查通常无特殊性。

## 4 CIP的影像学表现

怀疑CIP的患者，需行胸部CT检查（平扫或高分辨CT）。接受ICI治疗的患者，胸部CT出现新的非肿块性影像学改变，均需除外CIP。

CIP的肺损伤影像学表现模式呈多样性。以往的小样本研究及回顾性研究报道的影像学表现模式也有差别。CIP的影像学基础征象可包括磨玻璃影、实变、纤维条索、小叶间隔增厚、牵张性支扩、小结节影、网状影等^[7, 12]^。结合病理的影像学类型最常见的为机化性肺炎，其次可以表现为非特异性间质性肺炎（nonspecific interstitial pneumonia, NSIP）、弥漫性肺泡损伤/急性呼吸窘迫综合征（diffuse alveolar damage/acute respiratory distress symdrome, DAD/ARDs）、过敏性肺炎（hypersensitiviy pneumonitis, HP）等^[7, 12, 13]^。不同的影像学表现和疾病严重程度、对激素的敏感性及预后相关。表现为DAD/ARDS者可能病程进展快，对激素敏感性差，预后差。

CIP肺损伤的分布模式最常见为双侧多叶多段，也可见单侧或单叶病变。对于复发者影像学分布可前后一致，也可呈游走性^[7]^。

## 5 CIP的支气管镜表现

支气管镜用于明确诊断及除外感染。经支气管镜下深部取痰明确病原学有助于排除感染及指导抗菌治疗。支气管镜下肺泡灌洗液常可提示淋巴细胞炎症，支气管肺泡灌洗液（broncheoalveolar lavage fluid, BALF）细胞学常表现为淋巴细胞比例的增加。BALF的病原学结果可提示病原学证据，为抗生素的使用提供依据。

部分患者可通过支气管镜行TBLB肺活检，病理检查对于CIP的诊断起支持作用。

鉴于临床鉴别除外感染难度大，且难治性患者缺乏有效的推荐治疗手段，建议CIP患者在呼吸情况允许时尽可能早期对于2级及以上CIP患者行支气管镜检查，以获取可靠的诊断和鉴别诊断依据来指导治疗。

## 6 CIP的病理学表现

对CIP肺活检标本的病理结果了解甚少。大多数CIP的标本来自于支气管镜下肺活检，样本量小，病理结果也可能缺乏代表性。尽管如此，大部分的CIP患者病理报告表现为淋巴细胞浸润、肉芽肿性炎症、机化性肺炎等。一项对于20例CIP的支气管镜下活检病理结果提示，所有患者都具有不同程度的淋巴细胞浸润（主要为T细胞），7例有肉芽肿，8例有嗜酸细胞浸润（包括2例有肉芽肿者）；19例标本的CD4/CD8染色显示浸润的淋巴细胞以CD8^+^ T细胞为主^[14]^。Naidoo等^[12]^报道了11例CIP患者的病理模式（8例经支气管镜肺活检，2例经皮肺穿，1例楔形切除），发现其中4例为细胞性间质性肺炎，3例以机化性肺炎为主，1例表现为弥漫性肺泡损伤，3例无明显异常。其中3例间质性炎症浸润病变中同时存在肉芽肿，2例同时存在嗜酸性粒细胞浸润。然而，CIP患者组织标本中浸润的炎症细胞是否为特定细胞亚群，与抗肿瘤的T细胞是否同源，尚需要进一步研究。

## 7 CIP的诊断及鉴别诊断

接受免疫检查点抑制剂的患者，在治疗过程中出现新发的影像学表现，结合其临床表现，需要考虑CIP。最终的诊断需要排除其他疾病，包括如下。

### 7.1 肺部感染（包括细菌、病毒、结核、真菌、PCP等）

ICIs治疗后出现感染性肺炎的诊治在以往的指南中往往不被涉及，人们认为T细胞激活后不会带来感染风险的增加，然而，越来越多的临床实践以及近期的*meta*分析^[5]^均提示，接受PD-1/PD-L1的患者不仅有发生各种免疫性肺炎的风险，而且发生感染性肺炎的风险也大于化疗组/安慰剂组。然而，相对于CIP，目前肺部感染仍主要作为CIP的鉴别诊断加以鉴别。

患者合并发热、咳痰、血象升高等均可提示感染。阻塞性肺炎也是肺癌患者肺部感染的一种常见形式，其病原学以细菌为主。卡氏肺包囊虫感染可引起双肺磨玻璃影及低氧血症，病毒感染也可引起弥漫肺部病变。另外，接受ICIs患者出现真菌感染、真菌性气道炎、活动性肺结核等均有相关的个案报道^[15-17]^。肺部感染和CIP有时在影像学上不能简单鉴别，必须结合痰病原学、血清病原学，对有条件的患者行支气管镜深部留取标本进行鉴别。偶有CIP和肺炎不能完全鉴别、同时存在或CIP后继发感染者，均应在适当鉴别后给予经验性抗生素治疗，同时积极寻找病原学证据。另外，在CIP的治疗过程中，也要始终警惕因免疫抑制引起的继发机会性感染。

### 7.2 肿瘤进展及假进展

肿瘤进展引起的新发病灶，尤其是表现为癌性淋巴管炎者，临床表现为呼吸困难、咳嗽，影像学以多发小叶间隔增厚、多发微小结节为主要表现，以及某些肿瘤的假进展导致新发病灶者，都需要和CIP进行鉴别。

### 7.3 COPD急性加重

部分轻中度COPD未用药的患者在治疗期间可出现COPD的急性加重，影像学可表现为小叶中心性的小结节或细支气管炎，与CIP鉴别困难。因此用药前应对COPD患者进行肺功能评估，并予以分级治疗控制COPD。

### 7.4 放射性肺损伤

放射性肺炎最常发生在肺部放疗后2个月-6个月，病变大部分局限于放射野内，可伴或不伴呼吸道症状，症状可包括咳嗽、呼吸困难、低热等。偶见放射野外病变者，病理多为放疗后机化性肺炎，需要更长时间的激素治疗。

### 7.5 其他可引起呼吸困难、肺部影像学改变的病因

患者心功能不全导致肺水肿可引起呼吸困难，各种原因引起的肺泡出血以及肿瘤高凝导致肺栓塞等均可引起呼吸道症状，需和CIP鉴别。

### 7.6 其他免疫检查点抑制剂相关性毒副作用导致的呼吸道症状

如免疫相关性心肌炎导致心衰肺水肿、甲状腺炎引起甲状腺功能减低导致胸腔积液、肺水肿等引起低氧血症、免疫检查点抑制剂相关重症肌无力引起呼吸困难等，临床均需要和CIP鉴别。

## 8 CIP的分级

CIP的分级目前多按照影像学及临床症状两者之一或两者结合进行分类。但是在具体分级的时候缺乏一些特异性的指标。美国国家综合癌症网（National Comprehensive Cancer Network, NCCN）指南以临床结合影像学进行分级，具体标准为：1级：无症状；病变局限于一叶肺或病变范围 < 25%的肺实质；2级：出现新的呼吸道症状或原有症状加重，包括气短、咳嗽、胸痛、发热，以及所需吸氧条件升高；3级：症状严重，病变累及所有肺叶或 > 50%肺实质，日常活动受限；4级：危及生命的呼吸损害。然而该分级标准没有结合病情进展速度、病理损伤类型等，导致分级不一定能准确提示预后。临床上除了关注分级高的CIP以外，对于病情进展迅速、影像学提示可能为弥漫性肺泡损伤的这一类疾病，即使刚诊断时为2级-3级，也需要密切关注、及时处理并按更高等级的CIP进行治疗以改善预后。

## 9 CIP的治疗及注意事项

### 9.1 激素的治疗

免疫检查点抑制剂相关肺炎治疗的基本用药为激素，规律、足量的激素治疗可控制70%-80%的CIP^[1]^。

对于诊断明确的2级及以上CIP，具有临床症状者需开始激素治疗，1级CIP可暂时观察，但如果临床出现进展，则应开始激素治疗。

对于2级-3级的CIP，推荐使用1 mg/kg/d-2 mg/kg/d泼尼松的等效剂量激素治疗，可选择口服或静脉激素（泼尼松或甲强龙），对于更严重者或急性病程者，首选静脉激素。

对于激素的减量方法，推荐在观察到起始剂量（1 mg/kg/d-2 mg/kg/d）激素起效后（48 h-72 h），继续维持原剂量使用至7 d-14 d，随后开始逐步减量，控制整体疗程在6周-8周。一般不超过12周。足量激素治疗时间建议最长不超过3周。

激素使用过程中需要注意监测相应的毒副作用，嘱患者注意避免感染，监测血压、血糖、电解质等。由于大部分CIP的总体疗程在8周左右，起始剂量激素的疗程在2周左右，一般不需要常规预防性抗卡氏肺包囊虫治疗，但是20 mg/d的激素使用超过6周者，建议可加用预防PCP治疗。可常规补充钙剂、维生素D3。

### 9.2 激素不敏感CIP的治疗

CIP对于激素治疗是否敏感的判断时间在48 h-72 h，主要基于临床是否改善来判断，包括：一般情况是否好转，脏器功能是否稳定，呼吸困难、咳嗽有无改善，需要的吸氧条件有无降低，氧合指数有无降低等综合判断，可结合客观指标如指氧饱和度、血气分析，必要时复查胸部CT或胸片来判断。

对于初始激素治疗不敏感的CIP，考虑为难治性CIP。对于难治性CIP，首先应进一步鉴别诊断，进一步除外感染、肺栓塞等其他原因。对于呼吸条件允许者，应积极完善支气管镜检查。

治疗上，难治性CIP的治疗困难，目前尚无一致的推荐方案。根据既往的文献报道及临床实践，建议可考虑以下措施：①冲击量激素治疗：冲击量激素可发挥最大程度的糖皮质激素受体依赖作用和非受体依赖作用，具有极强的抗炎作用，在风湿性疾病的治疗中用于危及生命的重症或急症，如狼疮脑病、ANCA相关性血管炎引起的急进型肾炎等^[18]^。然而其副作用也明显增加，尤其是感染、消化道出血、水钠潴留及其他不常见的副作用。在CIP的治疗中，尚无指南推荐冲击剂量的激素，但是在急进性病程、CT表现为弥漫性肺泡损伤的患者，在能除外感染的情况下，理论上可以考虑激素的冲击治疗，但是具体的疗效如何尚需更多项研究的支持。具体用法为静脉甲强龙0.5 g/d-1.0 g/d，共3 d，后改为1 mg/kg/d的泼尼松或等效剂量的激素治疗。在用药前需要除外相应的禁忌证。用药同时注意补充钙剂，使用质子泵抑制剂，注意监测出入量及电解质。激素冲击前后建议患者进行适当隔离以减少感染的发生。②丙种免疫球蛋白（IVIG）：副作用少，通过被动免疫中和抗原起到抗炎作用，尤其适用于感染不能完全除外的患者。推荐的用法为IVIG每天20 g，连用3 d，或每天10 g连用5 d，必要时可重复使用。③IL-6受体抑制剂托珠单抗：该药物为强效的炎症因子IL-6抑制剂，尤其适用于合并系统性炎症反应综合征（systemic inflammatory response syndrome, SIRS）的患者，可阻断炎症瀑布反应，减少全身炎症反应和肺部损害，根据单臂研究^[19]^结果，托珠单抗和激素联合治疗3级-4级SAE的有效率达80%以上，但是其在CIP中的疗效尚需更多的数据评估。④TNF-α——英夫利西：抗肿瘤坏死因子抗体在多种指南中都被推荐用于肠炎、肾炎等患者，但是在肺炎中尚缺乏具有说服力的数据，少数个案或小样本的病例报道中曾有使用TNF-α治疗CIP者，但疗效不肯定^[7]^。而副作用方面，英夫利西抑制免疫，容易合并感染或导致潜伏性或慢性感染的活动或加重，如播散性肺结核、乙肝病毒活动等，因此在肺部感染较难除外的CIP患者中，英夫利西的使用尚需更多证据支持。⑤其他免疫抑制剂：包括吗替麦考酚酯、环磷酰胺等，均可用于免疫相关的肺间质性疾病，作为激素起效后的长期免疫抑制维持治疗。但其起效慢，对于急性病程的CIP治疗作用有限。

### 9.3 经验性抗生素的使用

在感染无法完全排除的CIP的治疗起始阶段，尤其是在急性进展性疾病的早期，感染经常较难排除，此时建议采用激素的同时加用经验性抗生素治疗。推荐按照社区获得性肺炎的抗菌治疗原则选择抗生素。对于临床、影像学或实验室检查提示特殊类型病原菌感染者推荐进行相应的抗生素治疗，同时积极寻找病原学的证据。对于肺内有阻塞性病变者，推荐按阻塞性肺炎的抗菌原则选择可以覆盖阳性菌及厌氧菌的广谱抗生素，有长期或反复住院病史者推荐抗菌谱覆盖铜绿假单胞菌。在治疗中应继续监测疗效并进一步除外感染，在感染除外、CIP控制的情况下，尽早停用广谱抗生素或进行抗生素降级治疗。

### 9.4 支持治疗

包括呼吸道支持、全身支持及合并症的处理。呼吸道支持治疗方面，CIP患者应根据呼吸及氧合情况选择合适的氧疗方式，同时注意痰液引流，加强呼吸道管理。支气管舒张剂及吸入激素适合存在基础慢性肺气道疾病者。给予适当的营养支持，保持足够的热量供给及蛋白质，同时注意控制血糖。

## 10 预后转归及再挑战

绝大部分CIP预后良好，在激素停药后可恢复良好，少数预后不好者，多与激素治疗不敏感、激素免疫抑制治疗后继发感染或肿瘤进展相关。

明确诊断的Ⅱ级及以上CIP患者，均需暂停ICI的治疗。在CIP治疗后，部分患者可考虑ICI的再挑战。最新的回顾性研究^[20]^提示，在93例出现irAEs的患者中，40例患者接受了再挑战，17例（42.5%）患者出现同一类型irAEs的复发，5例出现了新的irAEs，其中5例CIP患者再挑战后有1例出现了CIP复发，而且复发的irAEs没有较初发的irAEs更严重或更难治。

然而对于再挑战的具体原则并无定论，建议根据以下原则进行考量：①前期ICI治疗的疗效：前期ICI取得完全缓解者，建议观察；前期疾病进展者，不再考虑接受ICI治疗；前期ICI获得部分缓解或疾病稳定者，可考虑再挑战。②初发CIP的情况：对于1级-2级CIP，激素治疗敏感者，考虑再挑战；部分3级CIP，激素治疗敏感且恢复良好者，考虑再挑战；建议再挑战前复查肺功能（包括通气功能、容量及弥散功能的测定），评估初次CIP后肺功能受损的程度及后续对于再次出现CIP的耐受性。对于肺基础疾病严重、激素治疗后吸收缓慢、无法8周-12周内完全停药或一次CIP后肺功能受损严重者，不建议再挑战。

接受再挑战的患者，除了定期评估疗效外，应严密监测毒副作用，包括CIP以及其他irAEs，如再次出现CIP复发，则治疗后不再考虑再次挑战。

再挑战用药方面，如初始为PD-1/PD-L抑制剂联合CTLA-4双免疫检查点抑制剂治疗，因曾有报道^[21]^提示继续双免疫检查点抑制剂治疗复发的风险大且严重程度高，不建议继续联合治疗，可考虑选择其中一种再挑战。对于单免疫检查点抑制剂治疗者，一般选择原药再挑战，换药再挑战的风险及疗效如何，目前尚无更多数据支持。
